# Vaccination of HIV positive children... a genuine challenge

**DOI:** 10.1186/1471-2334-13-S1-O3

**Published:** 2013-12-16

**Authors:** Delia Adelina Vlad, Mariana Mărdărescu, Cristina Petre, Ruxandra Neagu-Drăghicenoiu, Rodica Ungurianu, Sorin Petrea, Ana Maria Tudor, Alina Cibea, Carina Matei, Odette Chirilă, Alexandra Mărdărescu, Mihai Mitran

**Affiliations:** 1 National Institute for Infectious Diseases “Prof. Dr. Matei Balş”, Bucharest, Romania; 2 Romanian HIV/AIDS Centre, National Institute for Infectious Diseases “Prof. Dr. Matei Balş”, Bucharest, Romania; 3 Clinical Hospital for Obstetrics and Gynecology “Prof. Dr. Panait Sârbu”, Bucharest, Romania

## Background

Increased vulnerability of HIV infected children to numerous other infections is an argument for vaccination protection. Lately, we have been facing diseases that can be prevented by vaccination in children, perinatally exposed to HIV.

## Methods

During 01 January 2012 – 01 September 2013, in the Pediatric and Adolescents Immunosuppression Department of the National Institute for Infectious Diseases “Prof. Dr. Matei Balş” we performed an analysis of the immunizations in children perinatally exposed to HIV, aged 0-18 months. Data from medical records and vaccination history refer to the vaccination share and track, namely from the maternity to the general practitioner. A major setback is parents’ reluctance in accessing medical services. Most of them expose various reasons for avoiding contact with the medical staff, so that most children do not benefit from prevention through vaccination.

## Results

A percentage of only 46.42% were vaccinated at birth. Only 21.42% were vaccinated for tuberculosis, prematurity representing a key factor for forestalling this type of vaccine. A single perinatally exposed to HIV child was vaccinated with the flu vaccine, optionally. We found an increased incidence of acute respiratory infection with RSV, rotavirus enterocolitis, often with severe evolution. Abandonment, poverty, lack of understanding, mothers who use drugs, represent, likewise, causes that impede children to HIV positive mothers to be immunized.

## Conclusion

Infants and children with HIV infection require protection against preventable diseases. Immunization does not influence HIV progression, but the lack of vaccination may lead to severe, potentially fatal infections.

